# Comprehensive Bioinformatics Analysis and Validation of the Mechanism of Glutamic-pyruvic Transaminase 2 in Bladder Cancer

**DOI:** 10.7150/jca.119178

**Published:** 2025-10-27

**Authors:** Haonan Dong, Hongqiong Li, Shi Chen, Qun Wang, Yawei Zhang, Hongjin Shi, Jieming Zuo, Junhao Chen, Jiansong Wang, Haifeng Wang

**Affiliations:** Yunnan Institute of Urology, The Second Affiliated Hospital of Kunming Medical University, Kunming 650101, China.

**Keywords:** bladder cancer, glutamine metabolism, drug resistance, epithelial-mesenchymal transition (EMT)

## Abstract

**Objective:** Glutamic pyruvic transaminase 2 (GPT2) promotes the initiation and progression of various cancers. However, its regulatory role in bladder cancer (BCa) remains unclear. In this study, we aimed to validate the role of GPT2 in BCa using bioinformatics analysis combined with *in vitro* and *in vivo* experiments.

**Methods:** We utilized bioinformatic approaches to download GPT2-related genomic datasets and preliminarily analyzed their expression profile and clinical significance in BCa. Multidimensional predictions regarding the mechanisms by which GPT2 influences BCa progression were generated by integrating diverse bioinformatic analyses. These predictions were further validated through *in vitro* and *in vivo* experiments to confirm GPT2 expression patterns and pro-tumorigenic mechanisms.

**Conclusion:** GPT2 is highly expressed in BCa and is associated with a poor prognosis in patients with BCa. GPT2 has been implicated in tumorigenesis, immune cell infiltration, cell proliferation, epithelial-mesenchymal transition (EMT), and maintenance of stemness. GPT2 knockdown reduced EMT and stemness in BCa cells, suppressed their proliferation, invasion, and migration, and inhibited subcutaneous tumor formation and growth in nude mice. Investigating and elucidating the mechanism of GPT2 in bladder cancer (BCa) provides novel evidence for further understanding the pathogenesis of bladder cancer and developing subsequent therapeutic strategies.

## Introduction

Bladder cancer (BCa) is among the most common malignancies of the urinary system. BCa is classified into muscle-invasive BCa (MIBC, 30%) and non-muscle-invasive BCa (NMIBC, 70%) based on the tumor infiltration depth^1^. Surgical resection remains the primary treatment for both MIBC and NMIBC. However, studies have reported high postoperative recurrence rates, frequent distant metastases, and poor prognoses in patients with BCa^2^.

The mechanisms underlying BCa metastasis are highly heterogeneous and a major focus of research. For instance, Cheng *et al.*^3^ demonstrated that the chimeric ribonucleic acid (RNA) SLC2A11 interacts with PTBP1 to promote BCa proliferation and metastasis. Xiao *et al.*^4^ revealed that ubiquitin-conjugating enzyme E2 S cooperates with tripartite motif-containing protein 21 to enhance lymph node metastasis in BCa. Wu *et al.*^5^ identified the SUMO E3 ligase mitochondrial E3 ubiquitin protein ligase 1 as a suppressor of lymphatic metastasis, which inhibits BCa lymph node spread by mediating mitochondrial HSPA9 translocation. These findings underscore the complexity of BCa metastasis and highlight the urgent need to identify novel targets associated with recurrence and metastasis for developing advanced therapeutic strategies.

Alterations in multiple metabolic pathways (including glucose, lipid, and amino acid metabolism) in cancer cells are collectively termed metabolic reprogramming^6^. Among these, dysregulated glutamine metabolism—reported in various cancers such as pancreatic, ovarian, and breast cancers^7^-^9^—is recognized as a phenomenon termed "glutamine addiction." Tumor cells rely on glutamine catabolism to generate α-ketoglutarate (α-KG), which fuels the tricarboxylic acid cycle and provides energy and biosynthetic precursors for cellular growth^10^. Additionally, glutamine metabolites regulate oncogenic signaling pathways to promote tumor progression. Thus, investigating metabolic reprogramming, particularly glutamine metabolism, is essential for understanding tumorigenesis.

Glutamic-pyruvic transaminase (GPT) plays a vital role in gluconeogenesis and amino acid metabolism across tissues, including skeletal muscle, kidney, and liver^11^. GPT1 is localized in the cytoplasm and is widely used as a clinical biomarker of liver disease. In contrast, GPT2 is more abundant than GPT1, particularly in the muscle and adipose tissues, suggesting its critical role in glucose, amino acid, and fatty acid metabolism and homeostasis^12^. GPT2 catalyzes the reversible reaction between glucose-derived pyruvate and glutamine-derived glutamate to produce alanine and α-KG, thereby promoting tumor growth and metastasis^13^. Elevated GPT2 expression is observed in proliferating breast cancer cells, and inhibition of GPT2 activity reduces cancer cell survival^14^. Furthermore, in PIK3CA-mutated colorectal cancer, GPT2-mediated generation of α-KG from glutamine is indispensable for cell growth^15^. All in all, GPT2 is a pivotal enzyme converting glutamine-derived glutamate into α-ketoglutarate (α-KG), had not been systematically investigated in BCa, despite its documented role in promoting tumor growth and anabolic metabolism in other malignancies. In this study, we aimed to employ integrated bioinformatics approaches combined with *in vitro* and *in vivo* functional experiments to predict and explore the potential mechanisms of action of GPT2 in BCa. Our findings aim to provide new insights into the molecular mechanisms underlying BCa progression.

## Materials and Methods

### Bioinformatics Analysis

Bladder cancer (BCa) RNA-seq data and associated clinical metadata were acquired via the UCSC Xena portal from The Cancer Genome Atlas (TCGA). Normal tissue transcriptomes were retrieved from the Genotype-Tissue Expression (GTEx) project, while cancer cell line expression profiles originated from the Cancer Cell Line Encyclopedia (CCLE). GPT2 expression values underwent log₂ transformation prior to visualization through box plots generated with the 'ggpubr' R package. Somatic mutation profiles of GPT2 in BCa were interrogated using cBioPortal (www.cbioportal.org). The R package 'mafTools' facilitated comparative mutation analysis between GPT2 high- and low-expression cohorts, with results presented as forest plots. For Gene Set Enrichment Analysis (GSEA), the 'clusterProfiler' package was implemented using Reactome pathways obtained from the Molecular Signatures Database (www.gsea-msigdb.org).

### Cell Culture

The human bladder cancer lines T24, 5637, and J82, along with immortalized urothelial SV-HUC-1 cells (all obtained from Cell Resource Center, Shanghai Institute of Life Sciences, CAS), were maintained under standard culture protocols. Cells were grown in manufacturer-specified media: DMEM (Gibco) for 5637 and J82, F-12K (Gibco) for SV-HUC-1, and RPMI-1640 (Gibco) for T24. All cultures contained 10% fetal bovine serum and 1% penicillin-streptomycin, and were incubated at 37 °C with 5% CO₂ humidification.

### Generation of Stable GPT2-Knockdown BCa Cells

Lentiviral vectors for GPT2 silencing (sh-GPT2) and empty vector controls (sh-Control) were purchased from KeyGEN BioTECH (Jiangsu, China). Cells were transduced with 20 μL viral supernatant per well and 5 μg/mL polybrene. Following lentiviral transduction, cells were cultured with 2 µg/mL puromycin (Gibco A1113802) for 7-10 days to select stably transduced pools. The medium containing puromycin was replaced every 2-3 days. Monoclonal stability was verified by continued culture under puromycin selection and confirmation of persistent GFP expression (for vectors containing GFP) sustained knockdown efficiency via qPCR and Western blot.

### Cell proliferation assay (cell counting kit [CCK-8])

Cells were plated in 96-well plates at 3,000 cells/well. At designated intervals (24, 48, 72, and 96 hours), 10 μL of CCK-8 solution (Beyotime), prepared at a 1:9 dilution in serum-free medium, was introduced. Following 2-hour incubations, optical density at 450 nm was recorded.

### Scratch wound healing assay

Confluent 6-well plate monolayers were scratched using a 1 mL pipette tip. After removing detached cells through washing, serum-free medium was added. Wound closure was assessed at 0h and 48h via inverted microscopy, with migration distances quantified using ImageJ.

### Cell cycle analysis

After 4 °C overnight fixation in 70% ethanol, cellular specimens underwent phosphate-buffered saline (PBS) washes followed by nuclear staining with propidium iodide (PI; Cat# C2015, Beyotime) via a commercial cell cycle kit (FXP018, 4Abio). Subsequent DNA content assessment employed flow cytometry (BD FACSCalibur), with analytical processing conducted in FlowJo (v10.8.1).

### Apoptosis assay

Cells were stained with Annexin V-FITC and PI (FXP018, 4Abio, China) following the manufacturer's protocol. Apoptotic rates were quantified using flow cytometry.

### Transwell migration or invasion assay

Migration: 4×10⁴ cells in serum-free medium were seeded into the upper chamber (Corning). A complete medium (800 μL) was added to the lower chamber. After 24 h, the migrated cells were fixed with 4% paraformaldehyde, stained with 0.1% crystal violet, and imaged (100×magnification).

Invasion: Matrigel (BD Biosciences) was diluted 1:8, coated onto Transwell inserts, and polymerized at 37℃. Cells were processed as previously described.

### Colony formation assay

Cells (1,000 /well) were seeded into 6-well plates. After 14 days, the colonies were fixed, stained with crystal violet, and counted.

### Tumor sphere assay

Cells (5,000/well) were cultured in ultra-low attachment plates with stem cell medium (DMEM/F12 containing 2×B27, 1×N2, 20 ng/mL epidermal growth factor, and 10 ng/mL fibroblast growth factor 2). Spheres (> 75 μm) were counted after 2 weeks.

### Immunohistochemistry

BCa tissue microarrays (HBlaU108Su01; Shanghai Xinchao Biotechnology) containing 68 tumors and 40 adjacent tissues were stained with an anti-GPT2 antibody. (proteintech 16757-1-AP) The staining intensity (0-3+) and positive cell percentage (0-4) were scored. Total scores (0-12) were categorized as negative (0), weak (1-4), moderate (5-8), or strong (9-12). The IHC scoring was performed independently by two experienced pathologists who were blinded to the clinical data. The inter-observer agreement was assessed using Cohen's kappa coefficient, which showed good consistency.

### Quantitative reverse transcription-polymerase chain reaction (qRT-PCR)

Following TRIzol (Invitrogen) RNA extraction, Total RNA (1 µg) was reverse-transcribed into cDNA using the Invitrogen SuperScript II (Thermofisher, 18064071) in a 20 µL reaction volume, according to the manufacturer's protocol. Quantitative PCR was then performed using Maxima SYBR Green/ROX qPCR (Thermofisher, K0223) on a LightCycler 480 II. The 10 µL PCR reaction mixture contained 5 µLMaxima SYBR Green/ROX qPCR, 0.5 µL each of forward and reverse primers (10 µM), 2 µL cDNA template, and 2 µL RNase-free water. The thermocycling conditions were as follows: 95 °C for 30 sec, followed by 40 cycles of 95 °C for 5 sec and 60 °C for 30 sec. GAPDH served as the endogenous control.

### Western blot

Proteins were isolated using RIPA lysis buffer, quantified by BCA assay, and resolved via SDS-PAGE. Transferred membranes were incubated with primary antibodies targeting GPT2(proteintech 16757-1-AP), E-cadherin (proteintech 20874-1-AP), N-cadherin (proteintech 22018-1-AP), Vimentin (proteintech 10366-1-AP), Snail1 (proteintech 13099-1-AP), ALDH1(proteintech 15910-1-AP), SOX2(proteintech 11064-1-AP), OCT4(proteintech 11263-1-AP), and KMT1A (proteintech 10574-1-AP), followed by HRP-conjugated secondary antibodies. Immunoreactive bands were detected by enhanced chemiluminescence (ECL). Use GAPDH as an internal reference, its molecular weight is 37kDa, which does not conflict with the size of the target protein.

### Xenograft tumor model

Thirty 5-week-old BALB/c nude mice (16-18 g) were injected subcutaneously with 6×10^6^ sh-Control or sh-GPT2 T24 cells (100 μL/mouse). Tumor volume (length×width²×π/6) and mouse weight were monitored every 3 days. The mice were euthanized at age 5 weeks, and the tumors were excised, weighed, and fixed in 4% paraformaldehyde. We defined humane endpoints, i.e. any mouse meeting one or more of the following criteria was euthanized immediately: Tumor burden: Tumor volume exceeding 1500 mm³; Body weight loss: A loss of more than 20% of the initial body weight; Ulceration or necrosis: Significant ulceration or necrosis of the tumor that impaired the animal's well-being; General health: Signs of severe distress, lethargy, inability to access food or water, or other conditions suggesting poor well-being as outlined in our approved animal protocol. Euthanasia Method: Mice were euthanized via cervical dislocation under deep isoflurane anesthesia (5% for induction, 2-3% for maintenance) to ensure a painless and humane procedure.

### Statistical analysis

Quantitative analysis of cell counts and Western blot band intensities was conducted using ImageJ. Results are expressed as mean ± standard deviation (SD; n=3 biological replicates. All functional experiments were performed with three independent biological replicates. Data are representative of three independent experiments (for representative images). Statistical evaluations employed SPSS (v25.0; IBM Corp.) and GraphPad Prism 7.0 (GraphPad Software, San Diego, CA, USA). Statistical analyses were performed using Student's t-test for comparisons between two groups and two-way ANOVA for multi-group comparisons. Pearson correlation analysis was applied to assess variable associations. Survival rates were analyzed using the log-rank test, while hazard ratios with 95% confidence intervals were calculated using Cox proportional hazards regression models. Data are presented as mean ± standard deviation, with statistical significance defined as p < 0.05.

## Results

### Differential expression of GPT2 in BCa vs. normal tissues

**Figure 1:**
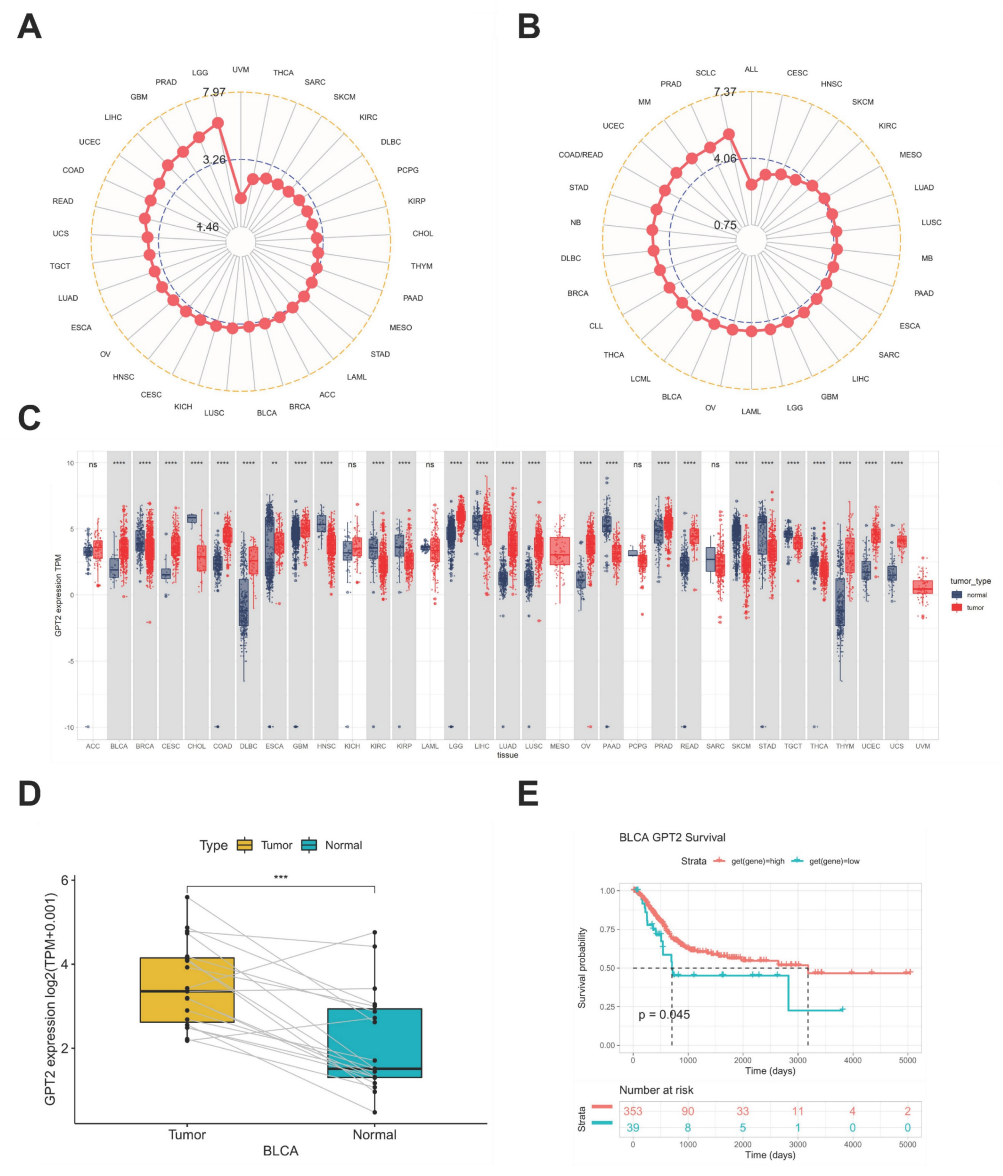
Analysis of GPT2 Expression and Prognostic Significance. (A) Expression levels of GPT2 in cancer cell lines. (B) Expression levels of GPT2 across 33 tumor types. (C) Comparison of GPT2 expression in tumor tissue cohorts. (D) GPT2 expression levels in paired tumor and adjacent normal tissues. (E) Survival analysis of TCGA patients stratified by high vs. low GPT2 expression. * *P* < 0.05, ** *P* < 0.01, *** P < 0.001.

Analysis of Cancer Cell Line Encyclopedia (CCLE) datasets showed widespread GPT2 overexpression across numerous cancer cell lines (Fig. 1a). Subsequent evaluation of The Cancer Genome Atlas (TCGA) data revealed elevated GPT2 levels in tumor tissues relative to matched normal samples (Fig. 1b), with 26 out of 33 tumor types exhibiting significantly higher expression (Fig. 1c). Paired bladder cancer (BCa) tissues displayed comparable overexpression patterns (Fig. 1d). Kaplan-Meier survival curves demonstrated significantly worse prognosis in patients with high GPT2 expression (Fig. 1e), collectively indicating GPT2 overexpression in BCa correlates with adverse clinical outcomes.

### Association between GPT2 expression, promoter methylation, and genetic mutations

**Figure 2:**
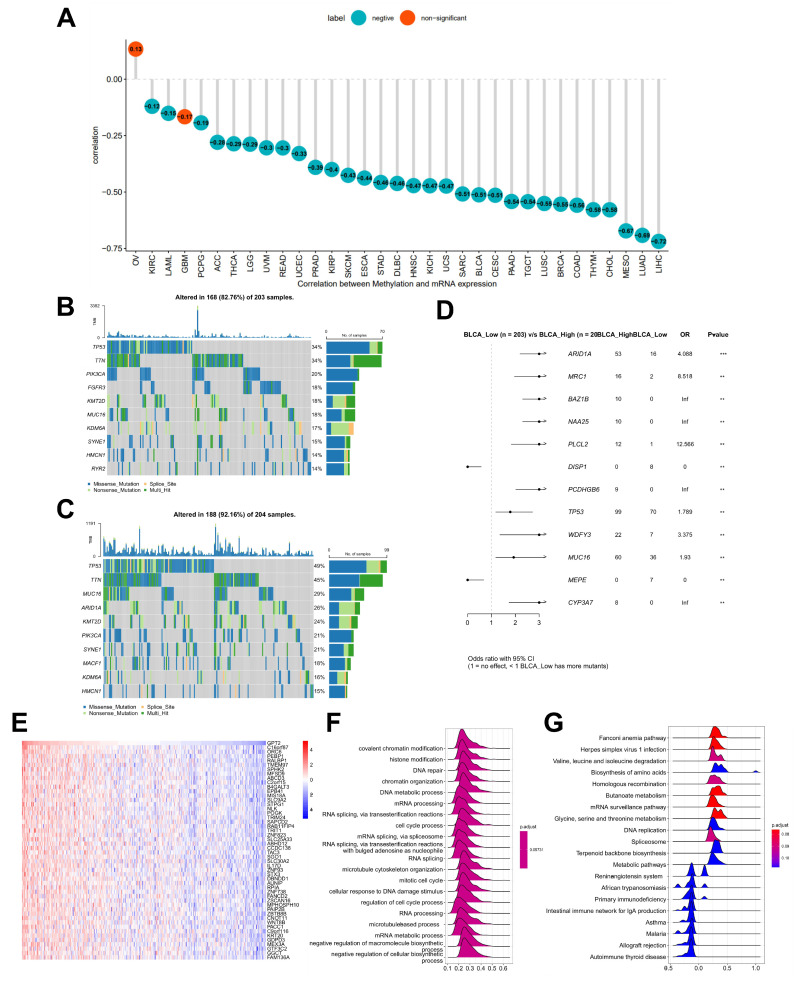
Promoter Methylation Features and Enrichment Analysis of GPT2. (A) Correlation between GPT2 expression and promoter methylation in pan-cancer tissues. (B) Gene mutations in BCa patients with low GPT2 expression. (C) Gene mutations in BCa patients with high GPT2 expression. (D) Comparison of mutated genes between GPT2 low-expression and high-expression groups in BCa. (E) Heatmap of the top 50 genes positively correlated with GPT2 in BCa. (F) GO enrichment analysis. (G) KEGG enrichment analysis.

Using the cBioPortal database, we explored the correlation between GPT2 expression and promoter methylation in 33 cancer types. The results showed a significant negative correlation between GPT2 expression and promoter methylation in most cancers, except ovarian cancer and glioma (Fig. 2a). Further analysis of GPT2-associated genetic mutations in BCa (Fig. 2b-c) identified ARID1A, MRC1, BAZ1B, NAA25, PLCL2, DISP1, PCDHGB6, and TP53 as significantly differentially mutated genes between GPT2 low- and high-expression subgroups (Fig. 2d). These results implied that GPT2 overexpression in BCa may result from promoter hypomethylation.

### Enrichment analysis of GPT2 and its correlation with tumor microenvironment (TME)

Correlation analysis between GPT2 and other genes revealed that the top 50 genes were most closely associated with GPT2 (heatmap in Fig. 2e). GSEA using 300 GPT2-positively correlated genes (Fig. 2f-g) indicated that GPT2 is predominantly involved in biological processes such as histone modification, DNA repair, messenger RNA processing, and cell cycle regulation, as well as pathways including amino acid metabolism, DNA replication, and immune response (we used an error detection rate < 0.25 and P adjustment < 0.05 as threshold parameters).

**Figure 3:**
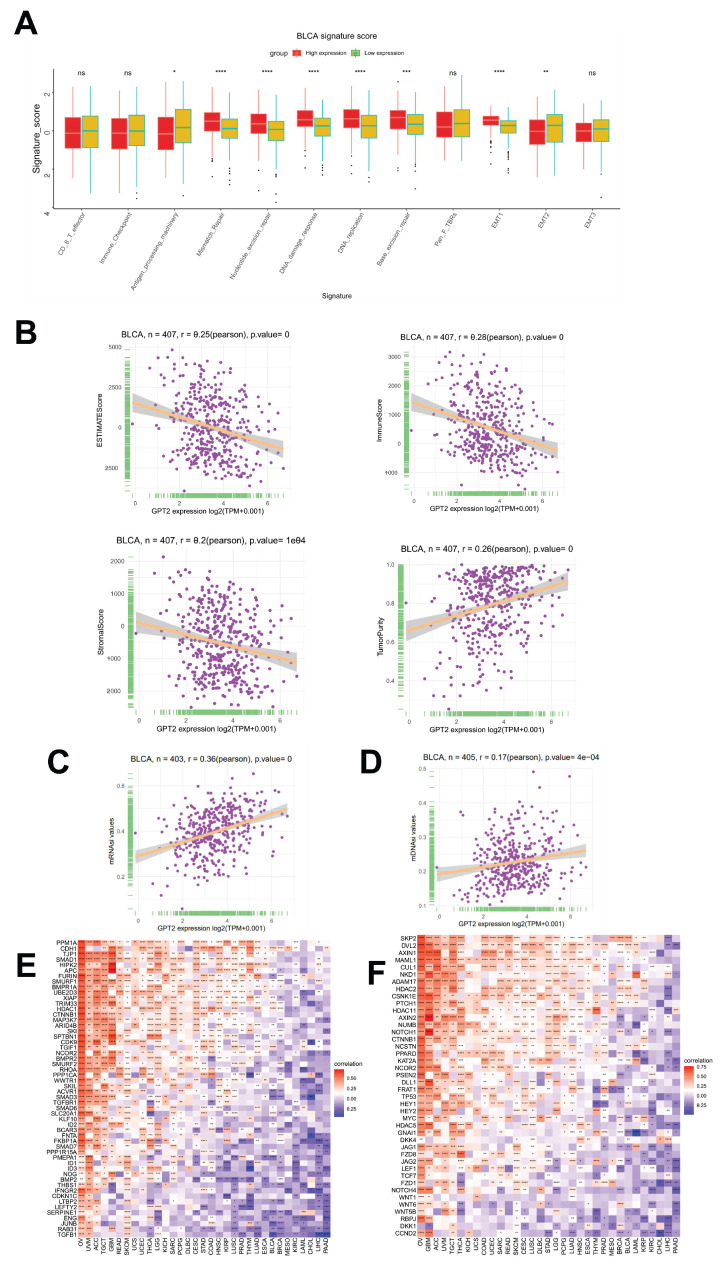
Correlation Analysis of GPT2 with Tumor Microenvironment and Stemness. (A) Stratification of GPT2 high- and low-expression groups based on distinct gene signatures (immune-related scores, mismatch repair-related scores, stromal-related scores, etc.). (B) Correlation between GPT2 expression and tumor purity, stromal score, immune score, and combined scores in bladder cancer. (C-D) Correlation of GPT2 with bladder cancer stemness indices: methylation stemness index (mDNAsi) and gene expression stemness index (mRNAsi). (E) Correlation between GPT2 expression and TGF-β1 signaling pathway genes. (F) Correlation between GPT2 expression and key genes in the Wnt/β-catenin signaling pathway.

The TME, which influences tumor proliferation, angiogenesis, invasion, metastasis, and chemoresistance, was further investigated for its GPT2-associated features. GPT2 expression significantly correlated with antigen processing, mismatch repair, nucleotide excision repair, DNA damage repair, DNA replication, base excision repair, and signatures (Fig. 3a). Additionally, using the ESTIMATE algorithm, we assessed the relationship between GPT2 expression and TME components in BCa, including tumor purity, immune score, stromal score, and combined scores (Fig. 3b). Notably, GPT2 expression was negatively correlated with immune, stromal, and combined scores, but positively correlated with tumor purity in BCa.

### Correlation analysis of GPT2 expression with tumor stemness

Cancer stem cells (CSCs) are a small subset of tumor cells with stem-like properties, including self-renewal and multilineage differentiation potential, collectively regarded as stemness. The gene expression stemness index for mRNA (mRNAsi) and DNA methylation stemness index (mDNAsi) are computational metrics derived from transcriptomic and methylation data, respectively. Both indices range from 0 to 1, with values closer to 1 indicating lower differentiation and stronger stem-like characteristics. We evaluated the correlation between GPT2 expression and BCa stemness using mRNAsi and mDNAsi (Fig. 3c-d). The results revealed a significant positive correlation between GPT2 expression and tumor stemness. Previous studies have highlighted the critical roles of the TGF-β1 and Wnt/β-catenin signaling pathways in regulating stemness and EMT across cancers. Gene co-expression analysis further demonstrated strong associations between GPT2 and key genes within the TGF-β1 and Wnt/β-catenin pathways (Fig. 3e-f). Heatmap analysis confirmed that GPT2 closely interacts with these pathways in multiple tumors, suggesting that GPT2 promotes tumorigenesis by modulating these signaling cascades.

### Correlation analysis of GPT2 expression with immune cell infiltration and immune-related genes

**Figure 4:**
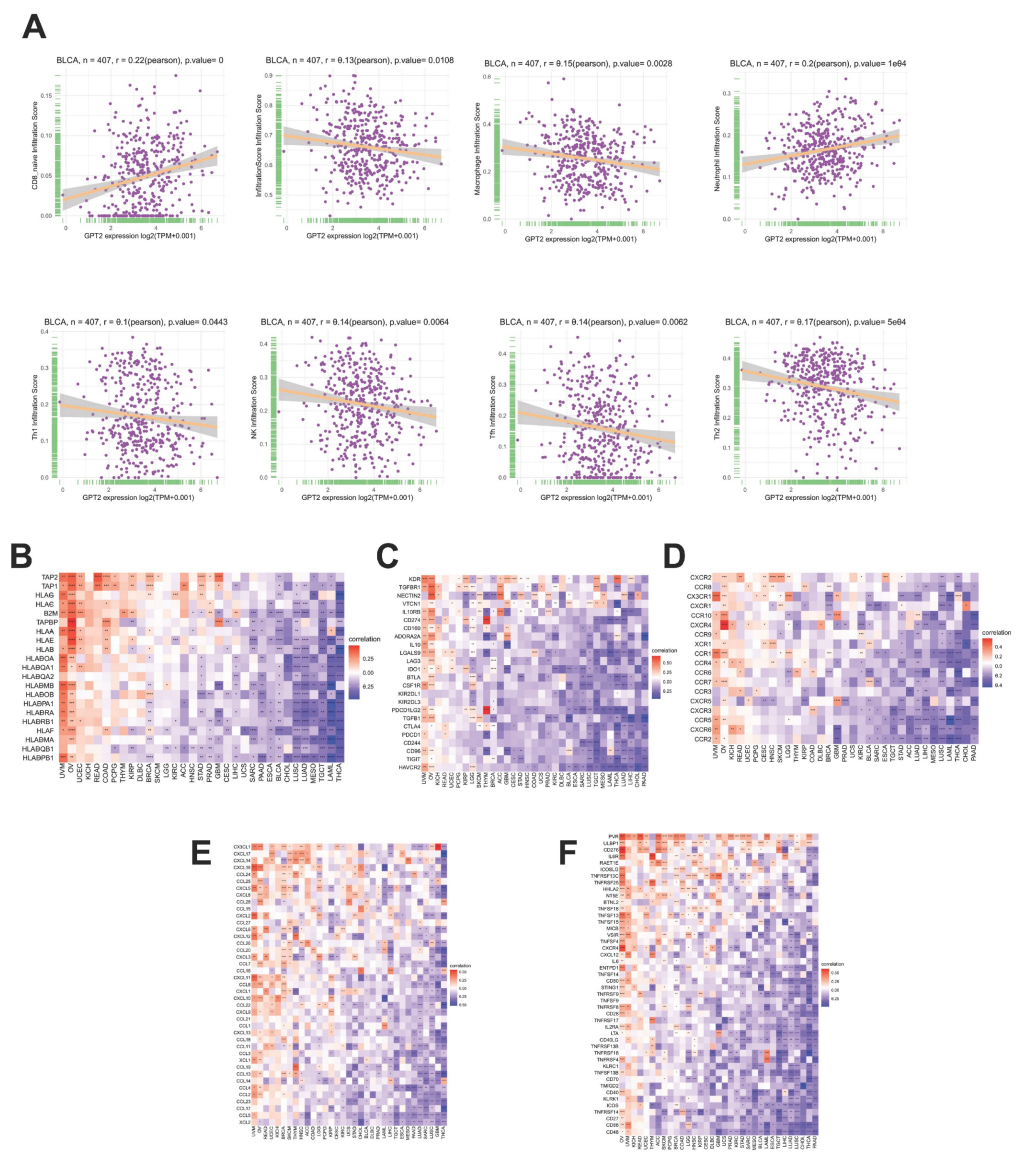
Association of GPT2 Expression with Immune Cell Infiltration and Immune-Related Genes in BCa. (A) Correlation analysis between GPT2 expression and infiltration of diverse immune cell subtypes in BCa. (B-F) Pan-cancer correlation of GPT2 with: (B) MHC genes; (C) Immunosuppressive genes; (D) Chemokines; (E) Chemokine receptors; (F) Immune-activating genes.

Using the ImmuCellAI database and CIBERSORT, we analyzed the relationship between GPT2 expression, immune infiltration scores, and 26 immune cell subtypes in BCa (Fig. 4a). In BCa, GPT2 expression was negatively correlated with overall immune infiltration scores. Specifically, GPT2 was negatively associated with macrophages, natural killer cells, Th2 cells, T follicular helper cells, and Th1 cells, except for CD8+ naïve T cells. Gene co-expression analysis was used to assess the correlation between GPT2 and immune-related genes, including major histocompatibility complex (MHC) molecules, immune activation or inhibition markers, chemokines, and chemokine receptors. Heatmap data (Fig. 4b-f) revealed that nearly all immune-related genes were co-expressed with GPT2, with most exhibiting negative correlations with BCa.

### Suppression of GPT2 induces cell cycle arrest and promotes apoptosis in BCa

**Figure 5:**
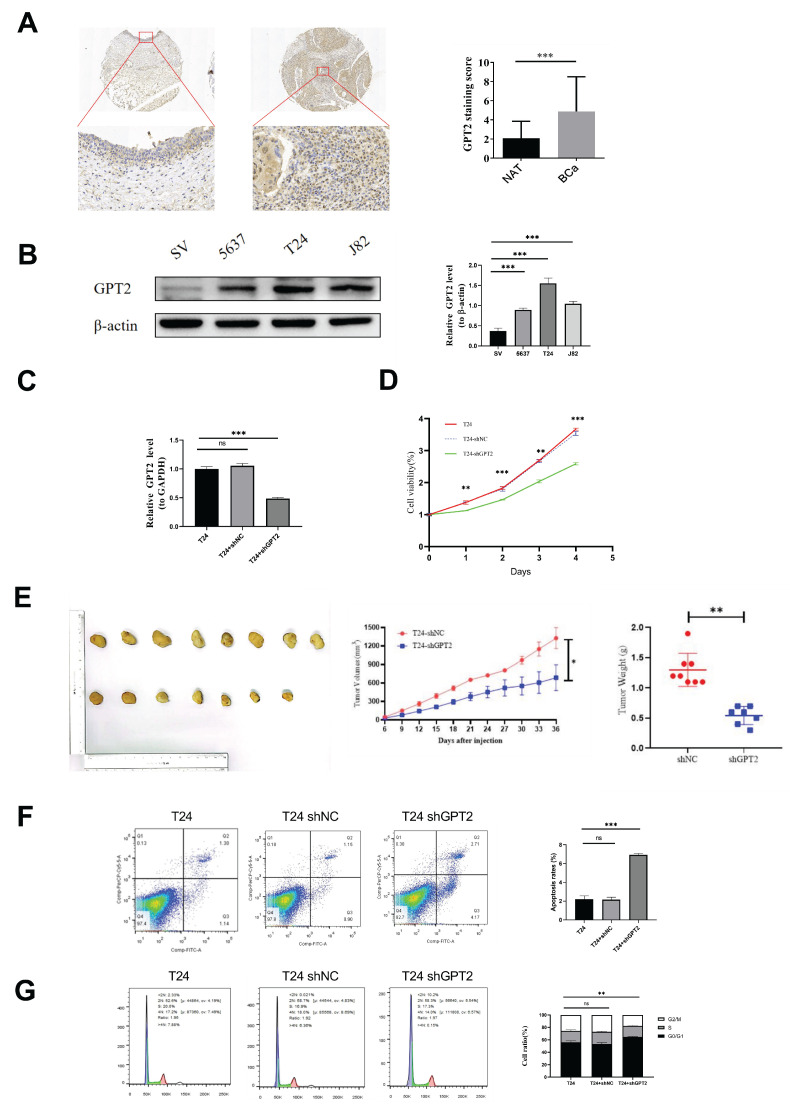
Knockdown of GPT2 Suppresses BCa Cell Proliferation *In Vitro* and *In Vivo*. (A) Immunohistochemistry (IHC) staining of GPT2 protein expression in bladder cancer tissues and adjacent normal tissues. (B) Western blot analysis of GPT2 expression in bladder cancer cell lines (5637, T24, J82) and normal urothelial cells (SV-HUC-1). (C) qRT-PCR validation of GPT2 knockdown efficiency in sh-GPT2 cells. (D) Cell proliferation assay following GPT2 knockdown. (E) Subcutaneous tumor formation assay in nude mice (right shoulder) with tumor size measurement and mass evaluation. (F-G) Flow cytometry analysis of (F) cell cycle distribution and (G) apoptosis rates in GPT2-knockdown cells.

Immunohistochemical staining of cancerous and adjacent tissues from 40 patients with BCa demonstrated significantly higher GPT2 expression in tumor tissues than in normal tissues (p = 0.0001, Fig. 5a). Western blot (WB) analysis of BCa cell lines (Fig. 5b) further validated this finding. GPT2 expression was markedly elevated in 5637, T24, and J82 cells compared to that in normal urothelial SV-HUC-1 cells (p < 0.001 for all), with the highest expression observed in T24 cells. Consequently, we established stable GPT2-knockdown T24 cell lines (sh-GPT2) and confirmed the knockdown efficiency using qPCR (Fig. 5c). Cell proliferation assays using these stable transfectants, along with wild-type T24 and empty vector controls, revealed that GPT2 suppression significantly inhibited BCa cell proliferation *in vitro* (p < 0.001, CCK-8 assay; Fig. 5d). Proliferation curves indicated a pronounced decline in the growth capacity following the GPT2 knockdown.

For *in vivo* validation, T24-ShNC (control) and T24-shGPT2 cells (6×10⁶ cells per injection) were subcutaneously injected into nude mice. Tumors formed in 8/8 mice injected with T24-ShNC cells within 2 weeks, whereas only 7/8 mice in the T24-shGPT2 group developed tumors. Tumor volume and mouse body weight were measured every 3 days from day 6 post-injection (tumor formation) for 4 weeks. At the endpoint, the tumors were excised and weighed (Fig. 5e). The sh-GPT2 group exhibited significantly slower tumor growth (p < 0.05) and reduced tumor mass (p < 0.01), confirming the anti-proliferative effect of GPT2 knockdown *in vivo*.

Flow cytometry analysis of stable transfectants and wild-type cells was used to assess cell cycle distribution and apoptosis rates. GPT2 knockdown increased the proportion of cells in the G0/G1 phase and decreased the proportion of cells in the S phase (Fig. 5f), indicating cell cycle arrest. Apoptosis assays showed that the apoptotic rate increased from 2% (control) to 7% in sh-GPT2 cells (Fig. 5g). The change was statistically significant (p < 0.05) and was consistent across biological replicates. This increase occurred alongside a more pronounced cell cycle arrest (a ~30% increase in G0/G1 phase fraction, Fig. 5F), indicating a combined anti-proliferative and pro-apoptotic effect. These results demonstrate that the targeted inhibition of GPT2 induces cell cycle arrest and promotes apoptosis in BCa cells.

### Knockdown of GPT2 inhibits EMT in BCa cells

**Figure 6:**
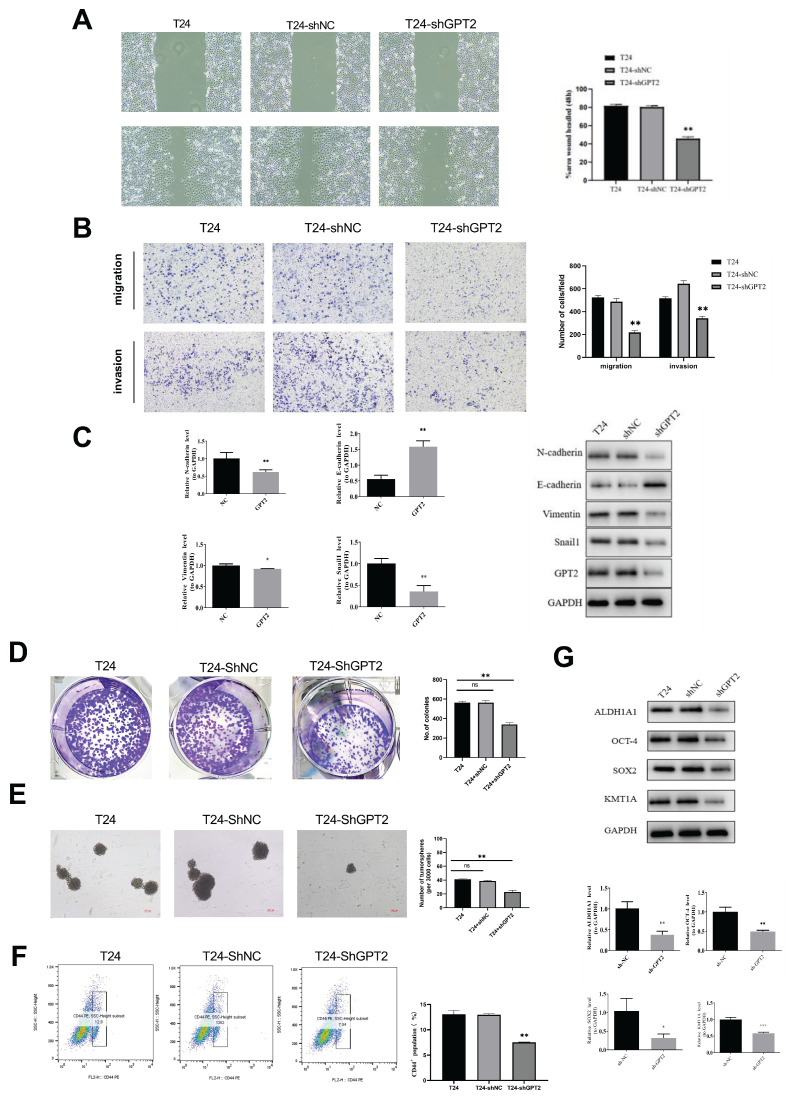
Knockdown of GPT2 Inhibits EMT and Downregulates Stemness Capabilities in Bladder Cancer Cells. (A) Scratch wound healing assay. (B) Transwell invasion assay. (C) qRT-PCR and Western blot (WB) analysis of EMT-related gene expression. (D) Colony formation assay of bladder cancer cells. (E) Tumor sphere formation assay of bladder cancer stem cells. (F) Flow cytometry analysis of CD44+ cell proportions. (G) qRT-PCR and WB analysis of stemness-related gene expression.

EMT is a biological process by which epithelial cells acquire mesenchymal phenotypes, enhancing tumor cell invasiveness and metastatic potential^16^. To investigate whether GPT2 influences EMT in BCa, we performed scratch wound healing and Transwell invasion assays. Results showed that GPT2 knockdown significantly reduced cell migration (p<0.01; Fig. 6a) and inhibited BCa cell invasiveness (p < 0.01; Fig. 6b). qRT-PCR and WB analyses further assessed GPT2- and EMT-related gene expression (Fig. 6c). GPT2 expression positively correlated with the epithelial marker E-cadherin and negatively correlated with mesenchymal markers (N-cadherin, Vimentin, and Snail1), suggesting that GPT2 suppresses BCa cell invasion and migration via EMT regulation.

### Knockdown of GPT2 downregulates stemness in BCa cells

As indicated by prior bioinformatics analyses, GPT2 expression is strongly correlated with tumor stemness. Tumor stem cells, which are characterized by their self-renewal and tumorigenic properties, are critical drivers of cancer initiation, recurrence, and metastasis^17^, ^18^. To validate these findings, we conducted colony formation and tumor sphere assays to evaluate the effects of GPT2 on cell stemness (Fig. 6d-e). GPT2 knockdown suppresses the sphere-forming ability and colony formation. CD44, a validated BCa stem cell marker positively associated with stemness, was analyzed using flow cytometry^19^, ^20^(Fig. 6f). The proportion of CD44+ cells decreased from 13% (control) to 7.54% in the GPT2-knockdown cells. Additionally, the mRNA and protein levels of stemness markers (ALDH1, SOX2, OCT4, and KMT1A) were decreased upon GPT2 suppression (Fig. 6g). These results demonstrated that targeting GPT2 attenuates stemness in BCa.

## Discussion

Metabolic reprogramming of glutamine and glucose is a hallmark of cancer^21^. GPT, an alanine transaminase, catalyzes the reversible transamination of alanine and α-KG to produce pyruvate and glutamate^22^. GPT plays critical roles in gluconeogenesis and amino acid metabolism in various tissues, including skeletal muscle, kidney, and liver^11^. GPT2 is vital for maintaining metabolic homeostasis of glucose, amino acids, and fatty acids^12^. We hypothesize that GPT2 maintains glucose metabolism via anaplerotic replenishment of TCA cycle intermediates (e.g., oxaloacetate and acetyl-CoA) and support of gluconeogenic pathways. This is achieved through its catalysis of pyruvate production from alanine, which fuels energy generation and biosynthetic processes. Additionally, GPT2 coordinates glutamine-derived carbon into the TCA cycle, facilitating metabolic flexibility in nutrient-scarce environments^23^-^25^. Glutamine dependence is a key feature of tumor growth and invasion. Elevated GPT2 expression is observed in proliferating breast cancer cells, and its inhibition reduces cancer cell survival^14^. In PIK3CA-mutated colorectal cancer, GPT2-derived α-KG from glutamine is essential for cell growth^15^. While GPT2 promotes tumor progression in breast cancer via GABA-mediated signaling pathways^10^, ^26^, its role in BCa may involve distinct mechanisms. In BCa, our data suggest GPT2 primarily drives EMT and stemness via glutamine-derived α-KG accumulation, which differentially modulates HIF-1α/Wnt pathways compared to other cancers. For instance, in breast cancer, GPT2-enriched exosomes activate BTRC to promote metastasis^27^. Analysis of the CCLE and TCGA datasets revealed GPT2 overexpression in multiple cancer cell lines and tumor tissues. Pan-cancer analysis further demonstrated significantly higher GPT2 expression in 26 cancer types, including BCa, than in adjacent normal tissues, and its overexpression was correlated with poor prognosis in BCa. Experimental validation confirmed the elevated GPT2 expression in BCa tissues and demonstrated that GPT2 knockdown suppressed proliferation, induced cell cycle arrest, and promoted apoptosis *in vitro* and *in vivo*. These findings align with bioinformatics predictions, underscoring the pro-tumorigenic role of GPT2 in BCa.

DNA methylation is a common epigenetic modification that regulates gene expression without altering the DNA sequence^28^. Aberrant methylation is implicated in cancer, aging, Alzheimer's disease, and other pathologies^29^. Hypermethylation of tumor suppressor genes and hypomethylation of oncogenes are hallmark features of cancer^30^. Our results revealed a strong negative correlation between GPT2 expression and DNA methylation in 31 cancer types, including BCa, suggesting that GPT2 overexpression in BCa stems from promoter hypomethylation. Further analysis of the GPT2 mutation profiles using cBioPortal identified TP53 as a frequently mutated gene in the high-GPT2 expression group, with ARID1A and TP53 being significantly differentially mutated between the groups. As tumor suppressors, TP53 mutations or deletions in cancers are linked to glutamine addiction. Mutant TP53 enhances glutamine metabolism by upregulating glutaminase 2, which enables tumor cells to tolerate glutamine deprivation and promote survival^31^. Similarly, ARID1A loss in ovarian cancer upregulates glutaminase 1, driving glutamine utilization^32^. These findings highlight TP53 and ARID1A as potential therapeutic targets for the inhibition of glutamine metabolism in BCa.

Metabolic reprogramming and immune evasion play a pivotal role in tumorigenesis^33^. Emerging evidence indicates that the remodeling of glutamine metabolism shapes antitumor immune responses within the TME^34^. Recent research has also proved this point, Hao *et al.* developed a glutamine metabolism-based prognostic model for MIBC, confirming GPT2's association with immune suppression and poor survival^35^. Our analysis showed that GPT2 expression negatively correlated with immune infiltration scores and most immune cell subtypes (except for naïve CD8+ T cells) in BCa. GPT2 is inversely associated with immune-related genes, including MHC molecules, immune checkpoints, and chemokines. Glutamine competition between tumor and immune cells in the TME critically affects antitumor immunity. For instance, glutamine-addicted clear cell renal carcinoma cells deplete extracellular glutamine, activating HIF-1α and inducing IL-23 secretion by tumor-associated macrophages, which amplifies regulatory T cell activity and suppresses effector T cell function^36^. Conversely, glutaminase-deficient tumors exhibit increased TME glutamine availability, which enhances T lymphocyte-mediated antitumor activity^37^. We hypothesize that high tumor GPT2 expression contributes to a glutamine-depleted TME, which primarily inhibits the function and infiltration of activated immune cells (leading to a lower overall immune score), while having a lesser impact on naïve cells. This interpretation, supported by the Krshnan's study^38^. Targeting glutamine metabolism to restore TME nutrient balance may thus potentiate immunotherapies.

GSEA of GPT2-correlated genes revealed enrichment in histone modification, amino acid metabolism, and DNA replication pathways. EMT, a key driver of tumor progression, was strongly linked to GPT2 expression in our TME analysis. EMT enables epithelial cells to acquire mesenchymal traits, enhancing invasiveness^39^. Stress induced by glutamine deprivation can trigger EMT in cancer cells^40^, suggesting that GPT2, a glutamine metabolic enzyme, may regulate this process. Co-expression analysis further implicated GPT2 in Wnt/β-catenin and TGF-β1 signaling, both central to EMT and stemness regulation^41^, ^42^. Experimental validation confirmed that GPT2 knockdown suppressed invasion or metastasis, upregulated E-cadherin expression, and downregulated N-cadherin, Vimentin, and Snail1, demonstrating the role of GPT2 in EMT and BCa progression.

Chemotherapy resistance in BCa has recently become a major focus of research. The first-line chemotherapy regimen for MIBC is primarily cisplatin-based. However, the objective response rate remains low (< 45%), and approximately 40% of patients experience postoperative recurrence^43^. Xie *et al.*^44^ also highlighted the prevalence of chemoresistance in BCa, demonstrating that targeting NAT10 significantly enhances chemosensitivity in CSCs, characterized by high tumorigenicity, multipotent differentiation, and resistance to radiotherapy and chemotherapy^45^, mediating tumor initiation, maintenance, and potential distant metastasis, leading to poor prognosis^46^. At the same time, bacterial outer membrane vesicles (OMVs) have also attracted researchers' attention because of their immune activation ability and potential as therapeutic agent carriers. Wang *et al.* Developed an engineered E. coli BL21 derived OMV platform (omv-c9112) based on crispr/dcas9, which can affect tumor chemotherapy resistance by delivering factors through this system^47^. Notably, EMT is prevalent in CSC populations and is driven by CSCs themselves^48^. Thus, CSC-targeted therapies represent a promising strategy for treating metastatic BCa.

In our study, GPT2 knockdown reduced the proportion of BCa stem cell subsets, suppressed cancer cell proliferation, and downregulated the CSC markers (ALDH1A1, SOX2, OCT4, and KMT1A). These findings suggest that GPT2 plays a critical role in maintaining stemness, offering a potential therapeutic avenue for overcoming chemoresistance and improving treatment outcomes. In conclusion, our findings demonstrate that GPT2 knockdown suppresses EMT, stemness, and BCa progression. However, the study had some limitations. First, the specific signaling pathways regulated by GPT2 require further elucidation. Second, the functional studies of GPT2 overexpression are needed to complement the knockdown data. Future studies should address these gaps to fully unravel the mechanistic contributions and therapeutic potential of GPT2 in BCa.

## Figures and Tables

**Table 1:** Primer sequences

Gene	Forward Primer (5'-3')	Reverse Primer (5'-3')
GAPDH	CCCATCACCATCTTCCAGG	CATCACGCCACAGTTTCCC
ALDH1A1	GCCAGGTAGAAGAAGGAGATAA	GTGGAGAGCAGTGAGAGGAGTT
OCT4	CAGAAGGGCAAGCGATCAAG	AGGGACCGAGGAGTACAGTG
NANOG	AATGGTGTGACGCAGGGATG	TGCACCAGGTCTGAGTGTTC
SOX2	CATGAAGGAGCACCCGGATT	TTCATGTGCGCGTAACTGTC
KLF4	GGACACACGGGATGATGCTC	GGACACACGGGATGATGCTC
KMT1A	GATCACCTGACAGACGGTGC	CCGTAACCACGTACAGCCAT
E-cadherin	TCCAGTGAACAACGATGGCA	CAACTGGAGAACGTTATTTTCTGT
N-cadherin	TATGGGAAATGGAAACTTGATGGC	TGGGTCTCTTTGTCTTGGGC
Vimentin	GGACCAGCTAACCAACGACA	AAGGTCAAGACGTGCCAGAG
Snail1	TAGCGAGTGGTTCTTCTGCG	AGGGCTGCTGGAAGGTAAAC
